# Knowledge, attitudes, practices (KAP), and risk factors toward zoonotic diseases among smallholder livestock farmers in Bugesera district of Rwanda

**DOI:** 10.3389/fpubh.2025.1569682

**Published:** 2025-04-17

**Authors:** Celestin Munyaneza, Ferdinand Bizimana, Felicitas Mukumbo, Sandrine Gatesi, Ephrem Sibomana, Severin Munyampuhwe, Marie Fausta Dutuze

**Affiliations:** Rwanda Institute for Conservation Agriculture (RICA), Bugesera, Rwanda

**Keywords:** smallholder livestock farmers, zoonoses, one health, knowledge, attitudes, practices, risk factors

## Abstract

**Background:**

Although zoonotic diseases pose significant health and economic threats globally, rural communities in developing countries are more vulnerable due to the increased proximity between animals and humans and the lack of knowledge about these diseases. This study assessed the knowledge, attitudes, practices (KAP), and risk factors regarding zoonotic diseases among smallholder livestock farmers in Bugesera district of Rwanda.

**Methods and materials:**

A convenient sample of 155 livestock smallholder farmers was selected from eight of the fifteen sectors of the district. Data were collected through interviews using a semi-structured questionnaire. Descriptive analyses including frequencies and means were used to summarize the data. Pearson’s chi-square test was used to examine associations between knowledge and socio-demographic variables and between knowledge and practices.

**Results:**

Findings showed that 50.3% of respondents knew diseases could be transmitted from animals to humans and just 13.5% recognized reverse zoonotic transmission - humans to animals. When specifically asked if they knew about brucellosis, tuberculosis, and Rift Valley fever; 88, 79, and 41% of respondents, respectively, reported being familiar with these diseases though many were unaware of their zoonotic nature. Risky attitudes and practices were prevalent, including the lack of isolation for sick animals (70.97%) and failure to quarantine newly introduced animals (83.87%). While 81.94% vaccinated their animals, only 16.54% could specify at least one vaccinated disease, and none knew the date of their animals’ next vaccination date. Other poor practices were reported, with 64.52% not separating animal and human utensils, and only 25.81% of cattle owners reported using artificial insemination. Additionally, 34.46% consumed raw non-boiled milk, and 24.5% did not use mosquito nets. Regarding roaming animals in the neighborhood, 79% of rats, 55% bats, 68% dogs, 67% cats, and 5.2% monkeys.

**Conclusion:**

The study revealed low awareness and high-risk practices regarding zoonotic diseases among smallholder livestock farmers in Bugesera district, posing a significant One Health concern. Therefore, educational programs to improve KAP and strengthen zoonotic disease prevention efforts in this district.

## Introduction

Livestock plays a crucial role in rural livelihoods and the economies of smallholder farmers in developing countries, including Rwanda (1, 2). In Rwanda, livestock accounts for 4% of the national Gross Domestic Product (GDP) and around 50% of private households own at least one animal (3). Most of these farm households are considered smallholder farmers and significantly contribute to the overall country’s livestock production. This population of smallholder farmers has limited economic resources and often lacks proper training in livestock management and formal education. They typically raise animals across generations, primarily for subsistence and cultural reasons, rather than for significant commercial purposes.

In Rwanda, owning livestock is driven by several reasons including economic and nutritional benefits to the households as well as manure production. In addition, cattle in particular holds cultural value and social significance (4). For instance, households’ prosperity is traditionally linked with the number of cows owned, cows are given as dowry in traditional wedding ceremonies, and cows and sometimes small ruminants are exchanged between friends to enhance social cohesion. While modernization is diminishing these practices in urban areas, they are still deeply rooted in rural areas. This strong connection between Rwandans and livestock increases the risk of zoonotic disease transmission, particularly in rural settings (5–7).

Several zoonotic diseases are reported in Rwanda including Rift Valley Fever (RVF), brucellosis, bovine tuberculosis, leptospirosis, rabies, anthrax, monkeypox, and Marburg. Rift Valley Fever is endemic with epizootic surges. A study conducted in the Eastern province showed RVF seroprevalence rates varying from 7.9 to 36.9% in cattle across all six districts (8). Severe RVF outbreaks occurred in 2018 and 2022 in both animal and human populations, with the Eastern and Southern provinces most affected (9–11). Brucellosis prevalence in cattle has ranged from 1.7 to 18.9% over the past decade (12). In goats, the prevalence of 10.7%. was reported (13). Bovine tuberculosis prevalence, as assessed at Nyabugogo abattoir was found to be 0.5% based on culture and postmortem results (14). A study on estimates of foodborne pathogens including *Campylobacter* spp., nontyphoidal *Salmonella enterica*, *Cryptosporidium* spp., *Brucella* spp., and *Mycobacterium bovis* due to consumption of raw milk and other dairy products, respectively, showed estimates of 71; 121; 12; 0.3; and 13 cases per 100.000 population (15). A study conducted on asymptomatic adult humans in Gisagara and Huye districts showed a high seroprevalence of leptospirosis of 40.1% (16). For rabies, although no research has been conducted to determine the prevalence, in 2016, 413 dog bites were documented leading to one human death (17). For anthrax, prevalence data is also limited, its presence was noted in both lowland and highland agro-ecological zones (18). Recently in 2024, Rwanda experienced monkeypox for the first time, primarily affecting rural areas with frequent human-animal interactions. Still in 2024, from September to December, Rwanda also faced the first outbreak of Marburg with 66 confirmed cases and 15 deaths. Furthermore, Rwanda shares borders with countries where other severe viral zoonotic hemorrhagic fevers including Ebola virus disease, Crimean-Congo hemorrhagic fever (CCHF), and Lassa fever have been reported such as Uganda, Democratic Republic of the Congo (DRC), and Tanzania (19–22).

Zoonotic diseases are associated with significant losses in both human and animal populations. The losses include sicknesses and/or deaths, reduced animal productivity, financial burdens of prevention, treatment, and control measures, and economic losses from trade bans or restrictions on the trade of animals and/or animal products (5, 23–28). These consequences are more exacerbated in local smallholder farmers who usually rely on their livestock as a main source of income and/or proteins from animal sources.

Many zoonotic diseases are considered important occupational health hazards among livestock keepers (5), as various management practices such as those implying direct contact with animals, handling aborted materials with bare hands, and consumption of unpasteurized-infected milk increase their exposure to these diseases (29).

Thus, for effective prevention of zoonotic diseases, it is essential to enhance knowledge and promote appropriate attitudes and practices, particularly among populations that have daily interactions with livestock. This approach would help mitigate zoonotic diseases as occupational hazards and reduce their prevalence in both human and animal populations (30). In this perspective, several studies have shown that a large number of livestock farmers are not aware of zoonotic diseases, and that some of their attitudes and practices expose them to the risk of zoonotic transmission (31, 32).

Although Rwanda has selected 6 priority zoonotic diseases in 2017 and strengthened their surveillance programs, these diseases remain a public health concern. These include (i) viral hemorrhagic fevers (Ebola, Marburg, Yellow fever, and CCHF), (ii) High Pathogenic Avian Influenza (HPAI), (iii) RVF, (iv) brucellosis, (v) sleeping sickness, and rabies (33, 34). Studies conducted in different districts of the country focused mainly on seroprevalence of these diseases (7–9, 12, 35–40). Very few studies were conducted on knowledge, attitudes, and practices (KAP) about these zoonotic diseases (41–43). The existing studies on KAP related to zoonotic diseases have focused on individual diseases, and thus did not provide a comprehensive status on KAP levels within the populations of study regarding the zoonotic diseases present in their environment.

The present study aimed to assess the level of KAP toward zoonotic diseases among smallholder livestock farmers in Bugesera district of Rwanda.

## Methods

### Study area

This study was conducted in Bugesera District, which is located in the Southwest part of the Eastern province of Rwanda. The district spans an area of 1,337 km^2^ and consists of 15 administrative sectors. In 2023, the population of Bugesera is estimated to be 551,103 inhabitants located in 137,777 households (44). The district belongs to the climatic zone of tropical savannahs with the annual rainfall variable ranging between 850 and 1,200 mm. Agriculture in Bugesera district is predominantly rain-fed, making it highly vulnerable to weather fluctuations. The district covers a total land area of 120,400 hectares, with approximately 72,300 hectares, or 60.05% of the total area, dedicated to agricultural use (45). Of 137,777 households, 59 and 42% are involved in crop and livestock farming, respectively. The households raising cows, goats, sheep, pigs, rabbits, chickens and other poultry are, respectively, 13.8, 24.0, 1.2, 8.3, 5.6, 14.5 and 1.1%. The number of cows, goat, sheep, pigs, rabbits, chicken, and other poultry are, respectively, 34,412; 100,332; 4,533; 24,459; 17,307; 313,761 and 5,439 (44).

### Study population

**Figure 1:**
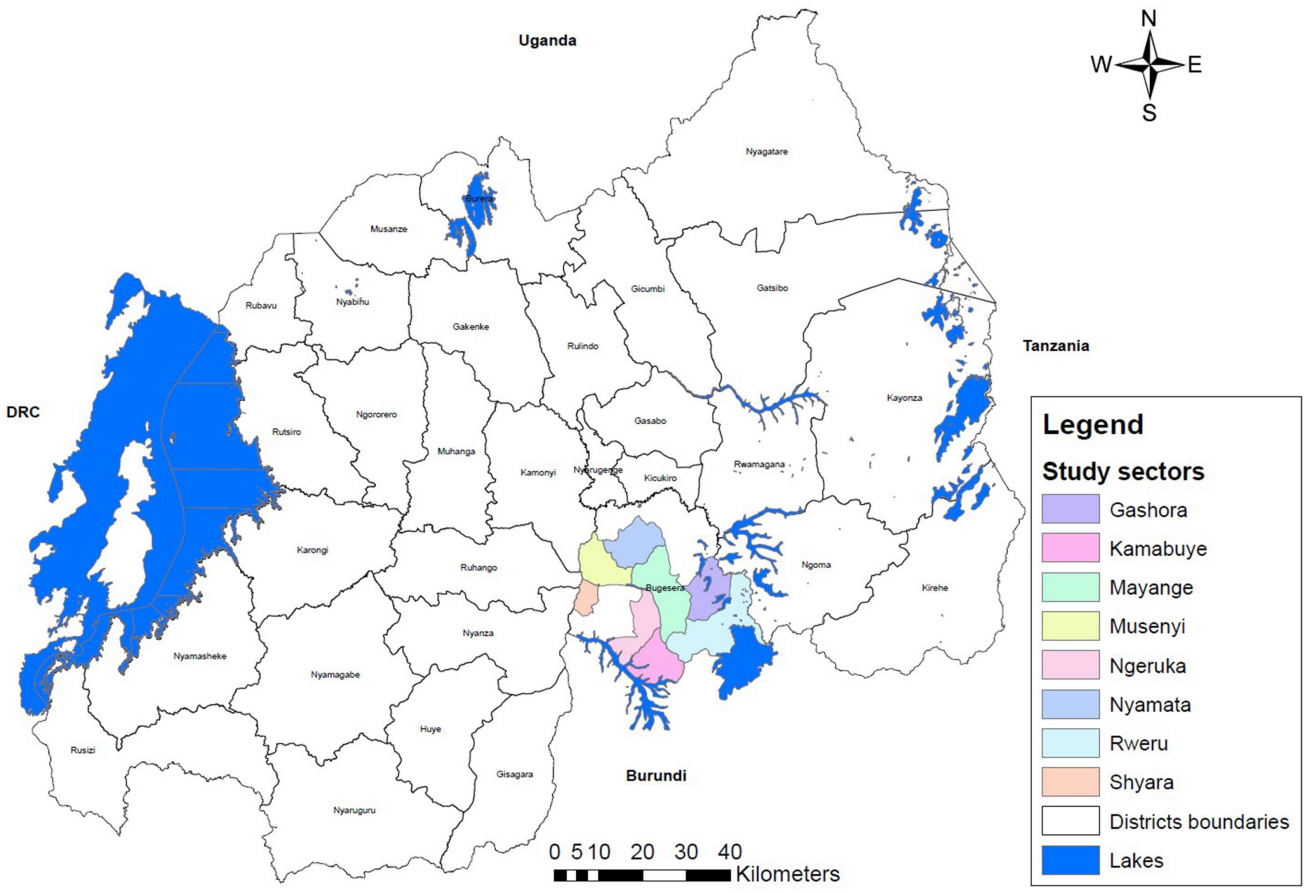
Map of the selected sectors of Bugesera district.

The study was conducted in 8 out of 15 administrative sectors of Bugesera District (Figure 1). These sectors are Gashora, Kamabuye, Mayange, Musenyi, Ngeruka, Nyamata, Rweru, and Shyara. The study population comprised of smallholder farmers who raise any type of domestic animal species namely cattle, goat, sheep, poultry, pig, and rabbit in the selected sectors. The sectors were selected based on the highest numbers of cattle population with the total cattle population. Cattle were considered as the reference species due to their significant role transmission of endemic zoonotic diseases such as Rift Valley fever (RVF), brucellosis, and tuberculosis, and high economic losses associated with them in cases of outbreaks (8, 9, 12, 14). In addition, its cultural value and its long-life cycle imply that cattle farmers usually keep the ‘farmer’ status for a long period as opposed to other species namely small ruminants, poultry, and pig farmers who are less consistent and abandon this business easily if productivity and production conditions are not optimal. Within the selected sectors, a convenient sample size of 155 smallholder farmers was determined based on financial and technical resources. A multi-stage sampling method was applied to a total of 49,011 households across the selected sectors—Gashora (5,131), Kamabuye (4,622), Mayange (6,617), Musenyi (7,123), Ngeruka (6,961), Nyamata (8,778), Rweru (6,399), and Shyara (3,380). The sample of 155 households was proportionally distributed among these sectors, with allocations of 15, 14, 19, 22, 21, 28, 24, and 12 households, respectively. Within each sector, households were selected randomly. The inclusion criteria consisted of belonging to a household that owns livestock in the same compound as humans and being an adult that was 18 years of age or older.

### Study design and data collection

A cross-sectional study was conducted in November 2023. The data were collected using a semi-structured questionnaire that was written in English and then translated in Kinyarwanda, with interviews conducted in Kinyarwanda. The questionnaire comprised diverse questions on farmer’s socio-demographic information, KAP, and risk factors associated with zoonotic diseases and it was pre-tested to ensure its validity.

### Data management and statistical analysis

The data obtained from the questionnaires were recorded using Microsoft Excel 2021. Statistical analysis was done using R 4.4.1 statistical software. Socio-demographic data were summarized using *gtsummary R* package. These were presented as frequencies and percentages. Pearson’s chi-square was used to assess the difference in levels of knowledge in different categories of socio-demographic characteristics. Additionally, the association between practices and knowledge was evaluated. Throughout the statistical analyses, statistical significance was considered significant if *p*-value ≤0.05.

### Ethical approval

The necessary ethical permission for conducting this study was obtained from the Rwanda National Research and Ethic Committee (RNEC) with identification number of IRB 00001973 of IORG0001100 – No 893/RNEC/2022. Participants gave their consent by signing a consent form before the interviews.

## Results

### Social demographic characteristics of the respondents

A little more than half (53%) of the respondents were females while the remaining were males. 5.2, 71, and 23.8% were, respectively, aged between 18 and 29, 30 and 60, and above 60 years. Regarding educational level, 29.9% did not have formal education, 55.6% had primary level, 13% secondary level, and 1.3% university level. The respondents had various experiences with livestock, namely, less than 5 years, 5 to 9 years, 10 to 19 years, and 20 and over years with the respective proportions of 21, 13, 33, and 33%. Most of the farmer households (83%) owned cattle, while 64, 43, and 32% kept goats, chickens, and pigs, respectively. A small proportion (7.7%) kept sheep and very few farmers kept rabbits and other poultry, including turkeys and ducks (5.8 and 3.9%, respectively). Few households kept dogs (7.10%) and cats (5.1%) (Table 1).

**Table 1:** Socio-demographic characteristics of the respondents.

Characteristic	Frequency (%), *n* = 155
Sector	
Gashora	15 (9.7%)
Kamabuye	14 (9.0%)
Mayange	19 (12%)
Musenyi	22 (14%)
Ngeruka	21 (14%)
Nyamata	28 (18%)
Rweru	24 (15%)
Shyara	12 (7.7%)
Gender	
Female	73 (47%)
Male	82 (53%)
Age categories (years)	
18–29	8 (5.2%)
30–60	110 (71%)
Above 60	37 (23.8%)
Education level	
None	46 (29.9%)
Primary	87 (55.6%)
Secondary	20 (13%)
University	2 (1.3%)
Marital status	
Married	128 (83%)
Single	27 (17.6%)
Experience raising animals (years)	
< 5	33 (21%)
5–9	20 (13%)
10–19	51 (33%)
>20	51 (33%)
Animals owned*	
Cattle	129 (83%)
Goats	99 (64%)
Sheep	12 (7.7%)
Pigs	50 (32%)
Chicken	66 (43%)
Rabbits	9 (5.8%)
Other poultry**	6 (3.9%)
Dogs	11 (7.1%)
Cats	8 (5.2%)
Number of cows (for cattle-owning households)	
No cattle	23 (14.8%)
1	43 (27.7%)
2–5	78 (50.3%)
>5	11 (7.1%)
Belonging to a farmer cooperative	
Yes	14 (9.0%)
No	
Participated in a training on livestock	
Yes	29 (19%)
No	
Visit	
Yes	72 (46%)
No	

*Respondents could own multiple animal species, **Other poultry species included turkeys and ducks.

Farmers mentioned various reasons for keeping livestock including the production of animal products such as milk, eggs, and meat for home consumption, selling animal products, selling live animals, saving money, production of manure, and others. Producing manure was mentioned as the main reason for keeping cattle (53.2%) among cattle keepers, followed by milk for home consumption (30.6%). The main reason for rearing goats and sheep was saving money with 42.7% of goat keepers and 63.6% of sheep keepers, respectively. Producing manure was the second reason for rearing goats and sheep with 32.2 and 27.2% of owner households, respectively. The two main reasons for rearing pigs were saving money and manure with 43.9 and 31.7% of households, respectively. The main reason for rearing chicken was for eggs for home consumption with 70.6% while 3.4% aimed at manure production. Rabbit keepers aimed at meat and manure production with the proportion of 42.8 and 28.5%, respectively. Cats and dogs were solely kept for protection.

### Knowledge of livestock farmers toward zoonotic diseases

As shown in Table 2, a little more than half of the respondents (50.3%) were aware that some diseases can be transmitted from animals to humans while only 13.5% of the respondents knew that diseases can be transmitted from humans to animals. 52.2% have heard of either diseases transmitted from animals to humans and/or humans to animals. Except for three respondents (1.9%), all those who acknowledged the possibility of human-to-animal disease transmission were also aware of animal-to-human transmission. Given their epidemiological importance in Rwanda, brucellosis, tuberculosis, and Rift Valley fever were specifically targeted by specific questions about them. To these questions, respondents showed levels of knowledge that were, respectively, of 88, 79, and 41%. Other diseases mentioned by respondents were gastro-intestinal diseases (3.8%), anthrax (2.6%), and rabies (1.3%). Due to the lack of knowledge, gastro-intestinal diseases were mentioned using broad terms without specifying diseases. The main sources of information included veterinarians (24%), radio (12%), other farmers (37%), local government officials (1.3%), schools (1.3%), and hospitals (1.3%).

**Table 2:** Knowledge of respondents about zoonotic diseases.

Question	Frequency (%), *n* = 155
Did you know that there exist diseases that can be transmitted from animals to humans?	78 (50.3%)
Did you know that there exist diseases that can be transmitted from humans to animals?	21 (13.5%)
*Diseases known*	
Have you heard of brucellosis?^1^	136 (88%)
Have you heard of tuberculosis?^1^	123 (79%)
Have you heard of RVF?^1^	63 (41%)
Other zoonotic diseases mentioned	
Anthrax	4 (2.6%)
Rabies	2 (1.3%)
Gastro-intestinal zoonotic diseases^2^	6 (3.8%)
*What is your main source of information?* ^3^	
Veterinarians	37 (24%)
Radio	19 (12%)
Other farmers	57 (37%)
Local government officials	2 (1.3%)
Schools	2 (1.3%)
Hospitals	2 (1.3%)

^1Specific questions about brucellosis, tuberculosis, and RVF were asked whether farmers have those diseases.^

^2Gastrointestinal zoonotic diseases were referenced using broad terms without specifying particular diseases.^

^3frequencies about sources of information are combinations of respondents who have heard about Brucellosis, Tuberculosis, and Rift Valley fever.^

### Factors influencing knowledge about zoonotic diseases

Table 3 indicates that factors such as sector, gender, age group, experience in livestock rearing, membership in a farmer cooperative, and visits from livestock professionals did not significantly influence knowledge levels about zoonotic diseases.

**Table 3:** Factors influencing knowledge about zoonotic diseases (*n* = 155).

	Have not heard of zoonotic diseases *	Have heard of zoonotic diseases*	Pearson’s chi-square *p*-value
Sector			
Gashora	6 (3.8%)	9 (5.8%)	0.6365
Kamabuye	7 (4.5%)	7 (4.5%)	
Mayange	11 (7%)	8 (5.1%)	
Musenyi	9 (5.8%)	13 (8.4%)	
Ngeruka	9 (5.8%)	12 (7.7%)	
Nyamata	17 (10%)	11 (7%)	
Rweru	9 (5.8%)	15 (9.6%)	
Shyara	7 (4.5%)	5 (3.2%)	
Gender			
Male	34 (21.9%)	48 (31%)	0.09549
Female	41 (26.4%)	32 (20.6%)	
Age			
18–29	6 (3.8%)	2 (1.3%)	
30–60	51 (30.7%)	59 (37.8%)	0.2937
Above 60	18 (11.6%)	19 (12.2%)	
Experience with rearing livestock (years)			
Less than 5	21 (13.5%)	12 (7.7%)	0.18
5–9	9 (5.8%)	11 (7%)	
10–19	25 (16.1%)	26 (16.7%)	
Over 20	20 (12.9%)	31 (20%)	
Belonging to a farmer cooperative			
Yes	5 (3.2%)	9 (5.8%)	0.4719
No	70 (45.1%)	71 (45.8%)	
Attended a farmer training			
Yes	15 (9.6%)	14 (9%)	0.8471
No	60 (38.7%)	66 (42.6%)	
Received a visit from a livestock professional			0.176
Yes	15 (9.6%)	14 (9%)	
No	60 (38.7%)	66 (42.6%)	

*Having heard of zoonotic disease is a combination of data for respondents who know about animal-to-human disease transmission and those who know about human–to–human transmission.

### Attitudes and practices that expose the farmers to the risk of zoonotic diseases

#### Attitudes and practices related to animal farm management

The majority of farmers (71 and 84.5%, respectively) do not isolate sick animals from healthy ones or quarantine newly introduced animals. While 82% reported vaccinating their animals, only 13.5% could name at least one vaccinated disease, and none knew the date of the next vaccination. Additionally, only 25.6% of cattle owners use artificial insemination. While nearly all farmers (99.3%) use farm manure, only 3.2% wear PPE, such as gloves, when handling it. Approximately 65.1% of respondents assist animals during parturition, but only 17.1% use protective measures, posing a potential risk of infection (Table 4). When asked about the disposal of abortive materials, 75% reported burying them, while 20% either discarded them in the bushes or fed them to dogs, and 5% disposed of them in toilets.

**Table 4:** Frequencies of respondents according to the practices associated with zoonotic disease transmission.

Question about practices/and other risk factors	Frequency (%), *n* = 155	
	No	Yes
Attitudes and practices related to animal farm management		
Do you isolate sick animals?	110 (71%)	55 (29%)
Do you practice quarantine of new animals?	131 (84.5%)	24 (15.5%)
Do you vaccinate your animals?	28 (18%)	127 (82%)
Can you mention at least one disease targeted during vaccination? (*n* = 127)^1^	110 (86.5%)	17 (13.5%)
Do you follow up with vaccination calendars? (*n* = 127)^1^	127 (100%)	0 (0%)
Do you use personal protective equipment (PPE) while handling manure? (*n* = 154)^2^	149 (96.8%)	5 (3.2%)
Do you use PPE while handling abortion? (*n* = 101)^3^	83 (82.2%)	18 (17.8%)
Do you practice artificial insemination? (for cattle owners only, *n* = 129)^4^	96 (74.4%)	33 (25.6%)
Attitudes and practices related to human lifestyle and human-animal interface		
Do you drink boiled milk?^5^ (*n* = 151)	55 (35.7%)	96 (62.3%)
If you do not drink mineral water, do you boil water before drinking it?	19 (70%)	46 (30%)
Do you eat undercooked meat?^6^ (*n* = 149)	92 (61.7%)	57 (38.2%)
Do you wash vegetables and fruits before eating them?	24 (15.6%)	131 (85%)
Do you separate animal and human equipment/ utensils?	100 (64.5%)	55 (35.5%)
Do your animals stay in animal housing during the night?	57 (36.8%)	98 (63.2%)
Do your animals stay in animal housing during the day?	62 (40%)	93 (60%)
Attitudes, practices, and risk factors associated with the environment		
Do you use mosquito nets?	38 (24.5%)	117 (75%)
Do you have bushes around you?	116 (74.8%)	39 (25.1%)
Is there any water body around you in a radius of 1 km?	118 (76.1%)	37 (23.8%)
Do you have the following roaming animals in your neighborhood?		
Dogs	49 (31.6%)	106 (68.4%)
Cats	51 (33%)	104 (67%)
Monkeys	8 (94.8%)	8 (5.2%)
Bats	69 (44.5%)	86 (55.5%)
Rats	33 (21%)	122 (79%)

^1 Only respondents who vaccinate animals responded to these questions.^

^2 Only respondents who use farm manure respondents to this question.^

^3 Only respondents who confirmed experiencing abortions on their farms responded to this question.^

^4 Only cattle owners responded to this question.^

^5^ Four (4) respondents indicated that they do not drink milk.

^6^ Six (6) respondents indicated that they do not eat meat.

#### Attitudes and practices related to human lifestyle and human-animal interface

As shown in Table 4, approximately 62.3% of respondents boil milk before drinking it. Although none of the respondents reported drinking mineral water, only 30% boil tap or lake water before consuming it. Additionally, 38.2% of the respondents who eat meat (149 individuals) reported consuming undercooked meat occasionally. Approximately 85% of respondents wash raw vegetables and/or fruits before consumption. However, more than half of the farmers (64.5%) do not separate animal and human utensils, including cleaning and kitchen utensils.

About 40% of cattle farmers did not have animal houses for daytime use, and 36.8% lacked houses for nighttime use. More animals were housed at night than during the day. Chickens were the most commonly housed animals, with 71.43% housed during the day and 73.33% at night, followed by cattle (70.59% during the day and 73.33% at night), and small ruminants (59.26% during the day and 76.92% at night). Alternative housing used at night included kitchens (20% of cattle, 100% of rabbits, and 76.92% of small ruminants), storage rooms for human use (11.5% of small ruminants), and human houses (75% of pigs and 11.54% of small ruminants). All dogs and cats were roaming freely around the houses during the day and night, occasionally sleeping in the kitchen.

#### Attitudes, practices, and risk factors associated with the environment

Regarding environmental risk factors, 75% of respondents reported using mosquito nets, while 24.8% live near bushes and 23.8% live less than 1 km from a water body. Additionally, respondents indicated the presence of roaming animals in their neighborhoods, including dogs (68.4%), cats (67%), monkeys (5.2%), bats (55.5%), and rats (79%) (Table 4).

### Relationships between knowledge and practices

Table 5 shows significant differences in knowledge about zoonotic diseases in relation to both vaccinating animals and boiling milk. A higher proportion of smallholder farmers who vaccinate their animals (45%) are aware of zoonotic diseases, compared to those who are not (5.8%). Similarly, a larger proportion of respondents who boil milk before drinking it (40%) have heard of zoonotic diseases, compared to those who have not (6.4%). No relationship was found between other practices and the level of knowledge about zoonotic diseases.

**Table 5:** Relation between knowledge and practices associated with zoonotic disease transmission.

	Do not know about zoonotic diseases*	Know about zoonotic diseases*	Pearson’s chi-square *p*-value
Do you practice quarantine?			0.3478
No	66 (42.5%)	65 (41.9%)	
Yes	9 (5.8%)	15 (9.7%)	
Do you vaccinate your animals?			0.03858
No	19 (12.2%)	9 (5.8%)	
Yes	56 (36.1%)	76 (45%)	
Do you PPE while handling manure?			0.403
No	73 (47.4%)	76 (49.3%)	
Yes	1 (0.6%)	4 (2.6%)	
Do you PPE while handling abortion? (*n* = 101)			0.3806
No	43 (42.5%)	44 (43.5%)	
Yes	6 (5.9%)	12 (1.8%)	
Do you drink boiled milk? (*n* = 151)			0.0008805
No	38 (25.1%)	10 (0.6%)	
Yes	35 (23.2%)	68 (44.1%)	
Do you drink boiled water?			0.1504
No	58 (37.4%)	51 (32.9%)	
Yes	17 (11%)	29 (18.7%)	
Do you eat undercooked meat? (*n* = 149)			0.1846
No	49 (32.9%)	43 (28.9%)	
Yes	22 (14.7%)	35 (23.5%)	
Do you wash vegetables and fruits before eating them?			0.2157
No	12 (7.7%)	10 (6.4%)	
Yes	61 (39.3%)	70 (45.1%)	
Do you separate animal and human equipment/ utensils?			0.9697
No	49 (31.6%)	51 (32.9%)	
Yes	26 (16.7%)	29 (18.7%)	
Do all your animals stay in animal housing during the day?			0.99
No	28 (18%)	29 (18.7%)	
Yes	47 (30.3%)	51 (32.9%)	
Do all your animals stay in animal housing during the day?			0.1007
No	36 (23.2%)	26 (16.7%)	
Yes	39 (25.1%)	54 (34.8%)	
Do you use mosquito nets?			0.1244
No	23 (14.8%)	15 (9.6%)	
Yes	52 (33.5%)	65 (41.9%)	

Expected where indicated otherwise, *n* = 155.

*Both respondents who know that diseases can be transmitted from animals to humans and those who know that diseases can be transmitted from humans to animals were categorized as ‘Know about zoonotic diseases’.

## Discussion

The demographic composition of the respondents, particularly the predominance of older and less-educated individuals, reflects the characteristics of smallholder livestock farming in Bugesera district. This should be taken into consideration while designing effective interventions to improve KAP regarding zoonotic diseases. The relatively small livestock numbers align with the typical smallholder farming systems, where farmers primarily raise animals for subsistence, cultural, and economic purposes. The findings also corroborate the Fifth Population and Housing Census of Rwanda conducted in 2012, which reported that most livestock farmers in Rwanda operate on a small scale and that sheep are relatively uncommon in the Eastern Province (44). As expected, cattle were found to be the most commonly owned animal species (83%), reflecting their significant cultural and economic importance to farmers. Beyond the historical preference for cattle compared to other livestock species in Rwanda, the number of cattle per household has increased due to the Girinka (One Cow per Poor Family) program, a social and agricultural initiative launched by the Rwandan government in 2006 (46). This program provides cows to vulnerable families to enhance nutrition, income, and agricultural productivity, with 450,000 cows distributed across the country as of 2021.

While Girinka has made important contributions to food security by improving nutrition, increasing agricultural productivity, reducing poverty, enhancing social cohesion - since beneficiaries are expected to pass on the first female calf to another vulnerable family- and promoting environmental sustainability (46, 47), it should also be accompanied by awareness campaigns on zoonotic diseases, given that cattle are main drivers of the most endemic zoonotic in Rwanda such as RVF, brucellosis, and tuberculosis (9, 11, 36, 48, 49).

A considerable proportion of the households kept livestock for their products, including milk, meat and eggs for sale and home consumption while other farmers raised livestock for producing manure. This interaction between human and domestic animals can influence attitudes and practices that expose livestock farmers to risks of contracting zoonotic diseases. Several studies have revealed that animal products such as milk, meat, eggs and manure can be a direct or indirect sources of zoonosis, if not handled or consumed correctly (50, 51). Although dogs and cats were raised for protection, they are known reservoirs of zoonotic diseases such as rabies, leptospirosis, salmonellosis, and leishmaniosis (52–56). This highlights the need for increased awareness about the possible zoonotic diseases that are related to different animal species owned by smallholder farmers, and appropriate biosecurity measures to mitigate the risk of zoonotic transmission.

Approximately half (50.3%) of the respondents demonstrated awareness of zoonotic disease transmission from animals to humans, but knowledge of reverse transmission (human-to-animal) was notably lower (13.5%). It was also found that except 3 respondents (1.9%), all respondents who know about human–to–animal transmission, are also aware of animal–to–human transmission. This may reveal that humans are generally concerned about human health and less about animal health, especially in rural areas where animal welfare is still not well known. 88,79, and 41% of respondents indicated having heard of brucellosis, tuberculosis, and RVF, however many of these are not aware of the zoonotic potential of these diseases. The increased awareness of these diseases may be attributed to their prevalence and economic impact in the region, as previous studies have documented significant seroprevalence rates for brucellosis (1.7–18.9%) (36, 57) and RVF (7.9–36.9%) (8, 9, 11) in Rwandan livestock populations. A prevalence of bovine tuberculosis in Nyabugogo abattoir was also found to be 0.5% (14). The more frequently diseases are reported in a geographic area, the greater the awareness among local inhabitants. When outbreaks occur - particularly those causing significant losses - they tend to receive high attention through various channels, including veterinarians, extension workers, radio broadcasts, and peer-to-peer farm communications. In addition, these three diseases are among the 6 selected priority zoonotic diseases at national level in 2017, alongside viral hemorrhagic fevers (Ebola, Marburg, Yellow fever, and CCHV), HPAI, and rabies (33, 34). This has increased the number of control programs specifically targeting them, which make them more familiar to the public ad especially to farmers.

Additional zoonotic diseases mentioned by respondents were anthrax (2.6%), rabies (1.35) and gastro-intestinal zoonotic diseases (3.8%). The low level of knowledge about anthrax and rabies is associated with low prevalence rates and limited research on them in Rwanda. Gastrointestinal zoonotic diseases were mentioned by respondents using general terms without specifying particular diseases. Under this group could be classified diseases caused by bacteria and parasites such as *Campylobacter* spp., *Salmonella* spp.*, Escherichia coli, Cryptosporidium* spp., *Toxoplasma gondii*, and others. Categorizing them using broad terms is a sign of lack of knowledge, which is a significant challenge since the diseases are usually associated with poor hygienic conditions which mostly characterize smallholder farms. In addition, poor practices associated with transmission of those diseases were reported such as failure to wash raw vegetables and fruits before consumption (15.6%), and failure to boil tap and/or lake water before drinking (70%).

The main sources of information were found to be other farmers (37%), veterinarians (24%), and radio (12%). This shows that educational programs such training of trainers (ToT) in which community leaders are educated to educate others can be effective. It is also positive to find that veterinarians constitute a reliable source of information, thus they can also plan more educational programs about zoonotic disease prevention. Although not specifically covered in this study, social media usage can be considered an important teaching channel due to its growing influence, along with the widespread coverage of mobile phones and the internet across the country. As of early 2025, mobile phone usage in Rwanda has continued to expand, reaching 92% of the population, while internet penetration accounts for 34.2% of the population (58).

Although no significant difference was observed, gender disparities in knowledge were observed, with men demonstrating slightly higher awareness of zoonotic disease transmission. This may be attributed to differences in access to information and involvement in livestock management activities. Similar trends have been found in a study conducted to determine the level of KAP regarding RVF in the eastern province which showed that 78.7% of male have heard of RVF as opposed to 59.7% in female population (41). Older individuals and those with more years of livestock experience were also more knowledgeable, likely due to accumulated exposure to farming risks and traditional knowledge. However, participation in farmer cooperatives and visits from livestock officers did not significantly influence knowledge levels, suggesting a need for more targeted and effective education programs.

Despite the potential health risks associated with livestock management, many farmer in the study did not implement critical biosecurity measures. The majority (71%) did not isolate sick animals, and 84.5% did not quarantine newly introduced animals, increasing the likelihood of disease spread within farms. Comparisons with other studies indicate that poor quarantine practices are common in pastoral communities in Africa, as reported in Kenya (59) and South Africa (60).

Vaccination coverage among farmers was relatively high at 82%, but very few (13.5%) could specify the diseases their animals were vaccinated against, and none were aware of the next vaccination date for their animals. This knowledge gap undermines the effectiveness of vaccination programs, as adherence to proper vaccination schedules is crucial for effective disease prevention. The discrepancy between high vaccination rates and the lack of commitment to following vaccination schedules may be attributed to the fact that for most major diseases, vaccination campaigns are conducted and subsidized by the government. As a result, farmers may not feel personally responsible for keeping track of or following up on their animals’ vaccination schedules. Strategies should be implemented to increase farmers’ accountability regarding animal vaccination. Additionally, artificial insemination use among cattle farmers was low (25.81%), a concerning trend given that natural breeding increases the risk of transmission of reproductive zoonoses such as brucellosis (50).

Risky food consumption behaviors were also observed, with 35.7% of respondents consuming unboiled milk and 61.7% eating undercooked meat. Given that the study population primarily consists of low-income smallholder farmers, consuming pasteurized milk may not be feasible; however, boiling milk before consumption is a practical and effective measure to prevent zoonotic transmission. Similarly, while 98.05% of respondents consumed vegetables, only 86.75% washed them before eating, increasing the risk of exposure to foodborne zoonotic bacteria and parasites such as *Campylobacter* spp., *Salmonella* spp.*, Escherichia coli, Cryptosporidium* spp., *Toxoplasma gondii*, and others (61).

Environmental factors play a crucial role in the transmission of zoonotic diseases. Approximately 75.48% of respondents in the study reported using mosquito nets, but a significant proportion lived near bushes (24.84%) or water bodies (23.86%). These environmental conditions increase the risk of exposure to vector-borne zoonotic diseases. While some of these diseases have already been reported in the area, others have not yet been detected, but the district is at risk due to its geographic features, such as low altitude, high temperatures, and the presence of numerous water bodies, all of which are favorable to the mosquito life cycle. Several zoonotic mosquito-borne diseases, such as Rift Valley Fever (RVF), which is endemic and causes severe outbreaks, as well as other bunyaviral infections like Bunyamwera and Batai, have been reported in Rwanda (9, 11), and other bunyaviral zoonotic infections such Bunyamwera, Batai, are reported in Rwanda (9). A study that aimed at determining occurrence of other arboviruses including chikungunya virus (CHIKV), o’nyong-nyong virus (ONNV), dengue virus (DENV), West Nile virus (WNV), Zika virus (ZIKV), Rift Valley fever virus (RVFV) and CCHFV showed that in a sample of 2,294 febrile human patients that were put in 230 pools, ONNV infection was detected in 12 pools (5.2%) while ZIKV was detected in three pools (1.3%). Other arboviruses were not detected in this study (62). Given that Rwanda is sharing borders with Uganda, Tanzania, and DRC where those diseases are reported, the risk of transmission is high.

A significant number of roaming animals, including rats (79%), bats (55.5%), stray dogs (68.4%), cats (67%), and monkeys (5.2%), were reported by respondents, posing a risk for zoonotic disease transmission. Rats serve as reservoirs for leptospirosis, salmonellosis, hantaviruses, toxoplasmosis, and plague. Cats can transmit toxoplasmosis, rabies, salmonellosis, campylobacteriosis, and leptospirosis. Stray dogs are potential carriers of rabies, leptospirosis, leishmaniasis, campylobacteriosis, and scabies. Monkeys contribute to the spread of rabies, Marburg virus disease, Ebola virus disease, monkeypox, yellow fever, tuberculosis, leptospirosis, and gastrointestinal parasites such as *Giardia, Cryptosporidium*, and *Entamoeba* spp. Bats are natural reservoirs of rabies, EVD, MVD, histoplasmosis, leptospirosis, severe acute respiratory syndrome (SARS), SARS-CoV-2 (COVID-19), and other coronaviruses (63–66). The presence of these animals significantly increases the risk of zoonotic disease outbreaks, especially since some of these diseases already reported in Rwanda.

Limitations of this study include the use of a convenience sample, which was determined based on available financial and technical resources, potentially affecting the generalizability of the findings. Additionally, some survey questions were structured as simple ‘Yes/No’ responses, whereas a Likert scale could have provided a more nuanced understanding of respondents’ behaviors. For example, questions such as *“Do you wash vegetables and fruits?,” “Do you boil milk before drinking?,”* and *“Do you boil water before drinking?”* assumed consistent practices, whereas a frequency-based scale (e.g., *always, often, sometimes, rarely, never*) would have better captured variations in behavior. This limitation may have led to an overestimation or underestimation of certain practices, impacting the accuracy of the study’s conclusions.

Overall, this study highlights significant gaps in KAP related to zoonotic diseases among smallholder livestock farmers in Bugesera district. The findings emphasize the urgent need for tailored educational programs focusing on disease awareness, biosecurity practices, and safe food handling. Strengthening vaccination programs, promoting AI adoption, and implementing stricter animal movement controls are also crucial measures to reduce zoonotic risks. Future research should include serological surveys of key zoonotic diseases among both livestock and human populations to better understand transmission dynamics and inform policy decisions.

## Conclusion and recommendations

Zoonotic diseases can have negative economic and social impact on livestock farmers and other stakeholders. This study assessed the knowledge, attitudes and practices among livestock keepers in Bugesera district. The results highlighted the crucial contributions of livestock toward the farmers’ livelihoods through the provision of food for family consumption, manure for fertilizing cropland, and generating income for daily or emergency expenses. However, some critical gaps in knowledge, attitudes and practices required to minimize the risk of zoonotic disease transmission were also revealed. This poses a risk to health, well-being and socio-economic stability in smallholder livestock farming communities.

The results from the study show that the knowledge about zoonotic diseases is still low. Some farmers do not know that a disease can be transmitted from animals to humans and vice versa. Others do not know the most endemic zoonotic diseases in Rwanda including brucellosis, RVF and toxoplasmosis. Farmers still have some attitudes and practices that expose them to risk of contracting zoonotic diseases. Those include drinking raw milk and undercooked meat, assisting animal parturition without protection, sharing utensils with animals, and staying in the same house with animals. Some preventions of zoonotic diseases are not respected. These include biosecurity measures such as vaccination and quarantine, using mosquito nets. Other factors can put livestock keepers to risk of zoonotic diseases including having roaming animals such as dogs, cats, bats and monkeys, having bushes around the house. Hence, there is a need for intervention to teach the farmers about zoonotic diseases and how they can be prevented. This study is crucial for guiding intervention. More studies are recommended, these include screening the most common diseases such as brucellosis, tuberculosis and RVF among the livestock keepers and their animals.

## Data Availability

The raw data supporting the conclusions of this article will be made available by the authors, without undue reservation.
