# Supplementary material for: Knowledge, attitudes, practices (KAP), and risk factors toward zoonotic diseases among smallholder livestock farmers in Bugesera district of Rwanda
Source: Front Public Health. 2025 Apr 17;13:1569682. doi: 10.3389/fpubh.2025.1569682 (PMC12043602; doi:10.3389/fpubh.2025.1569682)
Supplement: Supplementary file 1 [file Table_1.docx]

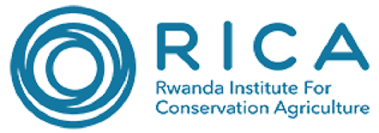


**Questionnaire/ *Ibibazo bijyana n’ubushakashatsi***

**IMPROVING KNOWLEDGE, ATTITUDES, AND PRACTICES (KAP) REGARDING ZOONOTIC DISEASES AMONG LIVESTOCK SMALLHOLDER FARMERS IN BUGESERA DISTRICT/ *KONGERA UBUMENYI N’IMIKORERE Y’ABAKORA UBWOROZI BUCIRITSE KU BIJYANYE NO KWIRINDA INDWARA ZIFATA ABANTU N’AMATUNGO***

***My name is ………, from RICA. Currently I am conducting my research which focuses on Knowledge and attitude on zoonotic diseases among smallholder dairy farmers. To manage this, I would like to ask you some questions. All information will be used for academic purpose and they are confidential.***

***If you agree, I am starting to ask the questions (Adjust this introduction to the consent form)***

| 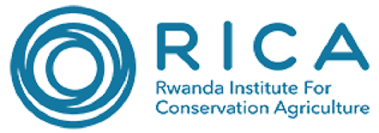 | **Research project:**  **IMPROVING KNOWLEDGE, ATTITUDES, AND PRACTICES (KAP) REGARDING ZOONOTIC DISEASES AMONG LIVESTOCK SMALLHOLDER FARMERS IN BUGESERA DISTRICT** |
| --- | --- |

**CONSENT FORM**

Greetings,

We are from the Rwanda Institute for Conservation Agriculture (RICA) and we are conducting a study on ‘*Improving Knowledge, Attitudes, and Practices (KAP) regarding zoonotic diseases among livestock smallholder farmers in Bugesera district’*. The overall objective of this study is to improve the livelihood of smallholder farmers in Bugesera by preventing zoonotic diseases in humans and animals in Rwanda.

Participation in this research is totally voluntary. If you agree to participate, we will ask you some questions related to your knowledge on diseases transmitted between animals and humans, your practices and interaction between the members of the household and your livestock.

We will further collect blood, milk, and fecal samples from some/or all of your livestock (cattle, goats, sheep, poultry, and pigs if any) from will which we will conduct laboratory analysis. In addition, we will collect blood sample from one adult person in your household for laboratory analysis as well. All those data will be put together for the determination of the status of zoonotic diseases in smallholder farmers in Bugesera.

**Benefits**: if you participate in this study, you will contribute to the improvement of human and animal health in Bugesera district and beyond. The results from this study will be communicated at district level.

**Your answers will be confidential**: The records of this study will be kept private. We will not include and personal information in any of our reports.

**Taking part is voluntary**: You may refuse to participate or stop participating at any time, and you may refuse to answer any question. Your decision not to participate, or to stop participating, will not affect you in any way.

If you have questions, you can contact Dr. Marie Fausta Dutuze on 0787363943 or Dr. Celestin Munyaneza on 0784643802.

Thank you for your time!

**Statement of Consent:** I have read the above information and I consent to take part in the study.

Your Signature ___________________________________ Date _________________________________

Your Name (printed) ____________________________________________________________________

| Sector.....................................................................Cell…………………........................................................ |
| --- |
| Telephone number…………………………………………………………………………………………………………………………… |

| 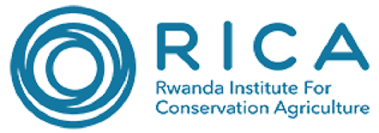 | **Ubushakashatsi:**  **KONGERA UBUMENYI N’IMIKORERE Y’ABAKORA UBWOROZI BUCIRITSE KU BIJYANYE NO KWIRINDA INDWARA ZIFATA ABANTU N’AMATUNGO MU KARERE KA BUGESERA** |
| --- | --- |

**AMASEZERANO YO KWEMERA KUGIRA URUHARE MU BUSHAKASHATSI**

Maramutse/ Muraho

Duturutse muri Kaminuza y’ u Rwanda yigisha ubuhinzi bubungabunga ibidukikije (RICA). Turi gukora ubushakasatsi ku ***‘Kongera ubumenyi n’imikorere y’abakora ubworozi buciritse ku bijyanye no kwirinda indwara zifata abantu n’amatungo mu karere ka Bugesera’.*** Ubu bushakashatsi bugamije guteza imbere imibereho y’abaturage tubafasha kwirinda indwara z’abantu n’iz’amatungo.

Gutanga umusanzu kuri ubu bushakashatsi si itegeko, ni amahitamo yanyu. Niba mubyemeye, turababaza ibibazo bitandukanye bijyanye n’ubumenyi mufite ku ndwara amatungo ashobora kwanduza abantu. Nyuma y’aho turabasaba gufata amaraso yanyu, amaraso y’amatungo yanyu, amata y’inka zanyu (niba mufite inka zikamwa) ndetse n’amase n’amatotoro y’amatungo yanyu. Ibi tuzabyifashisha dukora ibizamini byo muri laboratwari bijyana n’ubu bushakashatsi bigamije kureba niba mwebwe cyangwa amatungo yanyu afite izo ndwara abantu bahuriraho n’amatungo.

**Inyungu**: kwemera gufatanya natwe muri ubu bushakashatsi ni ugutanga umusanzu mu kumenya uko u Rwanda ruhagaze ku bijyanye n’indwara abantu banduzwa n’amatungo. Ibi bizatuma amategeko n’amabwiriza ajyanye no gupima indwara avugururwa maze kuvura no kwirinda indwara muri rusange bikarushaho gutanga umusaruro. Ibizava muri ubu bushakashatsi bizashyikirizwa ubuyobozi bw’akarere.

**Ibisubizo byanyu bizagirwa ibanga:** Amakuru muzatanga muri ubu bushakashatsi azaguma ari ibanga. Muri raporo zizasohoka muri ubu bushakashatsi nta makuru tuzashyiramo yatuma mumenyekana.

**Gukorana natwe ni ubushake**: Mushobora kwanga gukorana natwe cyangwa mukabihagarika igihe mushakiye ndetse ibibazo bimwe na bimwe ntimubisubize. Icyemezo cyanyu cyo gukorana natwe cg guhagarika gukorana natwe nta ngaruka zihariye kibagiraho.

Mugize ikibazo cyangwa mwifuje kugira ibindi bisobanuro mwahamagara Madame Marie Fausta Dutuze kuri No 0787363943 cyangwa Mr Celestin Munyaneza kuri No 0784643802.

Murakoze kutwemerera gufatanya natwe muri ubu bushakashatsi.

**Kwemera amasezerano:** Namaze gusoma ibyanditse mu masezerano, nemeye gufatanya namwe mu bushakashatsi.

Umukono ___________________________________ Italiki ____________________________________

Amazina (mu nyuguti nkuru) _____________________________________________________________

| Umurenge.....................................................................akagari…………………....................................... |
| --- |
| Numero ya telefoni………………………………………………………………………………………………………………………… |

**QUESTIONNAIRE INFORMATION/ *IBIJYANYE N’URUPAPURO RW’IBAZWA***

Questionnaire number/ *Numero*: ______________

GPS coordinates: Latitude (N/S): ……………. Longitude (W/E):………………….

Name of enumerator/ *Izina ry’umushakashatsi*: ___________________________________________________________

Date/ *Italiki*:______/______/2023

Starting time/ *Isaha ibazwa ryatangiriyeho*: ______:______

**A. FARMERS’ SOCIO-DEMOGRAPHIC INFORMATION/ *UMWIRONDORO W’UMWOROZI***

1. Sector/ *Umurenge*:_______________2. Cell/ *Akagari:* _____________3. Village/ *Umudugudu*: ____________________

4. Names of respondent*/ Izina ry’ubazwa*:___________________________ _____________________________________

5. Role in the household/ *Icyo ashinzwe & icyo ari cyo mu muryango*:

|  | Head of the household/ *Nyiri urugo* ( Man/ *Umugabo;* Woman/ *Umugore* ….. Child/ *Umwana*) |
| --- | --- |
|  | Spouse of the head of household/ *Umufasha wa nyir’urugo* |
|  | Child/ *Umwana* |
|  | Animal keeper/ *Umushumba* |
|  | Other (specify)/ *Ibindi (sobanura):* _______________________________________ |

6. Phone number/ *Numero ya telefoni*: 07____________________________

7. Sex/ *Igitsina*:_____________________ 8. Age of the respondent/ *Imyaka y’amavuko*:___________________________

|  | Male/ *Umugabo* | |
| --- | --- | --- |
|  | Female/ *Umugore* |  |

9. Formal education level/ *Amashuri (Tick below)* 10. Marital status/*Indangamimerere (Tick below)*

|  | None/*Ntiyize* |  |
| --- | --- | --- |
|  | Primary/ *Amashuri abanza* |  |
|  | Secondary 1/ *Icyiciro cya 1 cy’amashuri yisumbuye* | |
|  | Secondary 2/  *Icyiciro cya 2 cy’amashuri yisumbuye* |  |
|  | University/ *Kaminuza* |  |
|  | Other (specify)/ *Andi (Sobanura)* _____________ |  |
|  | ________________________________________ |  |

|  | Single/ *Ingaragu* |
| --- | --- |
|  | Married/ *Arubatse* |
|  | Widow/er/ *Yarapfakaye* |
|  | Other (specify)/ *Ibindi*  ___________________ |
|  | _______________________________________ |

11. Experience with livestock keeping/ *Imyaka amaze ari umworozi:* _________________________

12. What animal species do you have? / *Ni ayahe matungo ufite?*

|  | **Cattle**  ***Inka*** | **Goats**  ***Ihene*** | **Sheep**  ***Intama*** | **Pigs**  ***Ingurube*** | **Chickens**  ***Inkoko*** | **Rabbits**  ***Inkwavu*** | **Dogs**  ***Imbwa*** | **Cats**  ***Ipusi*** | **Others**  ***Izindi*** |
| --- | --- | --- | --- | --- | --- | --- | --- | --- | --- |
| ***Number & Breeds of animals/ Umubare n’ubwoko by’amatungo*** | | | | | | | | | |
| Total |  |  |  |  |  |  |  |  |  |
| Exotic/ *Inzungu* |  |  |  |  |  |  |  |  |  |
| Indigenous/ *Inyarwanda* |  |  |  |  |  |  |  |  |  |
| Crossed/ *Imvange* |  |  |  |  |  |  |  |  |  |
| ***Age & Sex categories/ Ibyiciro*** | | | | | | | | | |
| Female adults/ *Ingore nkuru* |  |  |  |  |  |  |  |  |  |
| Male adults/ *Ingabo nkuru* |  |  |  |  |  |  |  |  |  |
| Young males/ *Ibyana by’ibigabo* |  |  |  |  |  |  |  |  |  |
| Young females/ *Ibyana by’ibigore* |  |  |  |  |  |  |  |  |  |
| Milking cows ? (only for cattle) |  |  |  |  |  |  |  |  |  |
| ***Origin/ Aho yaturutse*** | | | | | | | | | |
| Bought/ *Zaraguzwe* |  |  |  |  |  |  |  |  |  |
| Born in the herd/ *Zikomoka mu rwuri* |  |  |  |  |  |  |  |  |  |
| From Girinka/ *Ni iza Girinka* |  |  |  |  |  |  |  |  |  |
| Gift from NGO/ *Zatanzwe n’umuryango ufasha* |  |  |  |  |  |  |  |  |  |
| Gift from another person/ *Impano y’undi muntu* |  |  |  |  |  |  |  |  |  |
| Other (specify)/*Ibindi* |  |  |  |  |  |  |  |  |  |
|  |  |  |  |  |  |  |  |  |  |
|  | **Cattle**  ***Inka*** | **Goats**  ***Ihene*** | **Sheep**  ***Intama*** | **Pigs**  ***Ingurube*** | **Chickens**  ***Inkoko*** | **Rabbits**  ***Inkwavu*** | **Dogs**  ***Imbwa*** | **Cats**  ***Ipusi*** | **Others**  ***Izindi*** |
| ***Main reasons for rearing animals/ Impamvu nyamukuru y’ubworozi (1; 2; 3)*** | | | | | | | | | |
| Selling milk/ *Kugurisha amata* |  |  |  |  |  |  |  |  |  |
| Milk for home consumption/ *Kubona amata yo kunywa mu rugo* |  |  |  |  |  |  |  |  |  |
| Selling meat/ *Kugurisha inyama* |  |  |  |  |  |  |  |  |  |
| Meat for home consumption/ *Kubona amata yo kunywa mu rugo* |  |  |  |  |  |  |  |  |  |
| Selling eggs/ *Kugurisha amagi* |  |  |  |  |  |  |  |  |  |
| Eggs for home consumption/ *Kubona amagi yo kurya mu rugo* |  |  |  |  |  |  |  |  |  |
| Selling young animals/ *Kugurisha into* |  |  |  |  |  |  |  |  |  |
| Selling adult animals/ *Kugurisha inkuru* |  |  |  |  |  |  |  |  |  |
| Bulls for insemination/ *Kubanguriza* |  |  |  |  |  |  |  |  |  |
| Saving money/ *Kubika amafaranga* |  |  |  |  |  |  |  |  |  |
| Leisure/ *Kwishimisha* |  |  |  |  |  |  |  |  |  |
| Protection/ *Uburinzi* |  |  |  |  |  |  |  |  |  |
| Other (specify)/ *Ibindi* |  |  |  |  |  |  |  |  |  |

13. Is any household member a member of one or more livestock farmers’ cooperatives? / *Hari umntu mu muryango wanyu uba muri koperative y’aborozi?*

|  | Yes/ *Yego* |
| --- | --- |
|  | No/*Oya* |

14. Have you/ any family member attended any training on livestock since last year? *Hari umuntu mu muryango wanyu wabonye amahugurwa y’ubworozi guhera umwaka ushize?*

|  | Yes/*Yego* |
| --- | --- |
|  | No/ *Oya* |

15. Have you had any livestock officer/ professional visiting your household since last year? *Hari umukozi ushinzwe iby’ubworozi wabasuye muri uyu mwaka cyangwa guhera umwaka ushize?*

|  | Yes/ *Yego* |
| --- | --- |
|  | No/ *Oya* |

**B. KNOWLEDGE, ATTITUDES AND PERCEPTION ON RISK OF ZOONOTIC DISEASES**

***UBUMENYI N’IMIKORERE KU BIJYANYE NO KWIRINDA INDWARA ZIFATA ABANTU N’AMATUNGO***

**B.1. KNOWLEDGE ON ZOONOTIC DISEASES**

16. Did you know that there exist diseases that can be transmitted from animal to humans? *Wari uzi ko hari indwara z’amatungo zishobora gufata abantu?*

|  | Yes/ *Yego* |
| --- | --- |
|  | No/ *Oya* |

17. Did you know that there exist diseases that can be transmitted from humans to animals? *Wari uzi ko hari indwara z’abantu zishobora gufata amatungo?*

|  | Yes/ *Yego* |
| --- | --- |
|  | No/ *Oya* |

*N.B: If the answer to N^o^ 16 and 17 is no go to question N^o^ 20*/ *Niba ibisubizo ari oya ku kibazo cya 16 n’icya 17, komereza ku kibazo cya 20.*

18. If the answer is yes to *N^o^* 16 and 17, which diseases do you know or have heard of? (Write 1, 2, 3.4,... for respectively the 1^st^, 2^nd^, 3^rd^, 4^th^…./ *Niba uzi ko zibaho, ni zihe waba uzi?(Andika 1, 2, 3, 4,… kuyo avuga ubwa 1, 2, 3,4,…)*

| **Disease** | **Humans**  ***Abantu*** | **Cattle**  ***Inka*** | **Goats & sheep**  ***Ihene & intama*** | **Poultry**  ***Inkoko*** | **Swine**  ***Ingurube*** | **Others**  ***Ibindi*** |
| --- | --- | --- | --- | --- | --- | --- |
| Brucellosis/ *Amakore* |  |  |  |  |  |  |
| Tuberculosis/ *Igituntu* |  |  |  |  |  |  |
| Toxoplasmosis/ *Togisopulasimozisi* |  |  |  |  |  |  |
| Anthrax/ *Ubutaka buterwa na Bacillus anthracis* |  |  |  |  |  |  |
| Black quarter/ *Ubutaka buterwa na Clostridium chauvoei* |  |  |  |  |  |  |
| Leptospirosis/ *Leputosipirozisi* |  |  |  |  |  |  |
| Salmonellosis/ *Salumoneloze* |  |  |  |  |  |  |
| Rift Valley fever/ *Ubuganga bwo mu kibaya cya Rift* |  |  |  |  |  |  |
| Rabies/ *Indwara y’ibisazi* |  |  |  |  |  |  |
| Dermatosis/ *Indwara z’uruhu* |  |  |  |  |  |  |
| Cysticercosis/ *rushe* |  |  |  |  |  |  |
| Others (specify)/ *Izindi* |  |  |  |  |  |  |
| Others (specify)/ *Izindi* |  |  |  |  |  |  |
| Others (specify)/ *Izindi* |  |  |  |  |  |  |

20. Disease specific questions:

|  | **Brucellosis/ *Amakore*** | **Tuberculosis / *Igituntu*** | **Rift Valley fever/ *Ubuganga bwo mu kibaya cya Rift*** |
| --- | --- | --- | --- |
| Have you heard of this disease?/ *Waba warumvishije iyi ndwara?* | \|  \| Yes/ *Yego* \| \| --- \| --- \| \|  \| No/ *Oya* \| | \|  \| Yes/ *Yego* \| \| --- \| --- \| \|  \| No/ *Oya* \| | \|  \| Yes/ *Yego* \| \| --- \| --- \| \|  \| No/ *Oya* \| |
| Main source of information/ *Wayumvise he?* | \|  \| School/ *Ishuri* \| \| --- \| --- \| \|  \| Training on livestock/ *Amahugurwa ku bworozi* \| \|  \| Radio & TV/ *Radiyo & Televiziyo* \| \|  \| Other farmers/ *Abandi borozi* \| \|  \| Other (specify)/ *Ibindi* _________________________ \| | \|  \| School/ *Ishuri* \| \| --- \| --- \| \|  \| Training on livestock/ *Amahugurwa ku bworozi* \| \|  \| Radio & TV/ *Radiyo & Televiziyo* \| \|  \| Other farmers/ *Abandi borozi* \| \|  \| Other (specify)/ *Ibindi* _________________________ \| | \|  \| School/ *Ishuri* \| \| --- \| --- \| \|  \| Training on livestock/ *Amahugurwa ku bworozi* \| \|  \| Radio & TV/ *Radiyo & Televiziyo* \| \|  \| Other farmers/ *Abandi borozi* \| \|  \| Other (specify)/ *Ibindi* _________________________ \| |
| What animal symptoms do you know?/ *Ibimenyetso mu matungo waba uyiziho ni ibihe ?* | \|  \| Abortion/*Kuramburura* \| \| --- \| --- \| \|  \| Stillbirth / *Kubyara inyana zipfuye* \| \|  \| Decreased milk production/ *Kugabanyuka k’umukamo* \| \|  \| Weight loss/ Kunanuka \| \|  \| Other (specify)/ *Ibindi* _________________________ \| \|  \| Other (specify)/ *Ibindi* _________________________ \| \|  \| Other (specify)/ *Ibindi* _________________________ \| | \|  \| Coughing/ *Gukorora* \| \| --- \| --- \| \|  \| Loss of appetite/ *Kwanda kurya* \| \|  \| Weight loss/ *Kunanuka* \| \|  \| Fluctuating fever/ *Umuriro uhindagurika* \| \|  \| Other (specify)/ *Ibindi* _________________________ \| \|  \| Other (specify)/ *Ibindi* _________________________ \| \|  \| Other (specify)/ *Ibindi* _________________________ \| | \|  \| Abortion/ *Kuramburura* \| \| --- \| --- \| \|  \| Weakness/ *Gucika intege* \| \|  \| Hemorrhage/ *Kuva amaraso mu myenge yose* \| \|  \| High death rate in a heard/ *Imfu nyinshi mu rwuri* \| \|  \| Other (specify)/ *Ibindi* _________________________ \| \|  \| Other (specify)/ *Ibindi* _________________________ \| \|  \| Other (specify)/ *Ibindi* _________________________ \| |
| What transmission modes do you know (to humans)?/*Waba uzi uko yandura mu bantu?* | \|  \| Contact with infected animals/ *Kwegera amatungo arwaye* \| \| --- \| --- \| \|  \| Drinking contaminated milk/cheese/ *Kunywa amata cg ibiyakomokaho byanduye* \| \|  \| Eating contaminated meat/ *Kurya inyama zanduye* \| \|  \| Other (specify)/ *Ibindi* _________________________ \| \|  \| Other (specify)/ *Ibindi* _________________________ \| | \|  \| Animal cough/sneeze/ *Kwegera itungo rirwaye riri gukorora* \| \| --- \| --- \| \|  \| Drinking contaminated milk/cheese/ *Kunya amata cg ibiyakomokaho byanduye* \| \|  \| Eating contaminated meat/ *Kurya inyama zanduye* \| \|  \| Other (specify)/ *Ibindi* _________________________ \| \|  \| Other (specify)/ *Ibindi* _________________________ \| | \|  \| Contact with infected animals/ *Kwegera amatungo arwaye* \| \| --- \| --- \| \|  \| Contact with abortion tissues/ *Gukora ku birambu* \| \|  \| Contact with blood/ *Gukora mu maraso yavuye ku matungo yanduye* \| \|  \| Mosquitoes/ *Kurumwa n’imibu* \| \|  \| Other (specify)/ *Ibindi* _________________________ \| \|  \| Other (specify)/ *Ibindi* _________________________ \| |
| Do you think you are at risk of getting this disease/ *Wumva iyi ndwara ushobora kuyandura* | \|  \| Yes/ *Yego* \| \| --- \| --- \| \|  \| No/ *Oya* \| | \|  \| Yes/ *Yego* \| \| --- \| --- \| \|  \| No/ *Oya* \| | \|  \| Yes/ *Yego* \| \| --- \| --- \| \|  \| No/ *Oya* \| |
| According to you, how can the disease be prevented/ *Ni gute iyi ndwara yakwirindwa?* | \|  \| Boiling milk/ Kunywa amata atetse \| \| --- \| --- \| \|  \| Eat well cooked meat/ *Kurya inyama zitetse neza* \| \|  \| Avoid direct contact with infected animals/ *Kwirinda gukora ku matungo arwaye* \| \|  \| Other (specify)/ *Ibindi* _________________________ \| \|  \| Other (specify)/ *Ibindi* _________________________ \| \|  \| Other (specify)/ *Ibindi* _________________________ \| | \|  \| Boiling milk/ *Kunywa amata atetse* \| \| --- \| --- \| \|  \| Eat well cooked meat/ *Kurya inyama zitetse neza* \| \|  \| Avoid direct contact with infected animals/ *Kwirinda gukora ku matungo arwaye* \| \|  \| Other (specify)/ *Ibindi* _________________________ \| \|  \| Other (specify)/ *Ibindi* _________________________ \| \|  \| Other (specify)/ *Ibindi* _________________________ \| | \|  \| Avoid contact with infected animals/ *Kwirinda gukora ku matungo arwaye* \| \| --- \| --- \| \|  \| Avoid contact with abortive tissues/ *Kwirinda gukora ku birambu*  Avoid contact with blood/ *Kwirinda gukora ku maraso yavuye ku matungo arwaye* \| \|  \| Other (specify)/ *Ibindi* _________________________ \| \|  \| Other (specify)/ *Ibindi* _________________________ \| \|  \| Other (specify)/ *Ibindi* _________________________ \| |

**B.2. ATTITUDES/ PRACTICES ASSOCIATED WITH RISK FACTORS/ *IMIKORERE IJYANYE NO KWIRINDA INDWARA ZIFATA ABANTU N’AMATUNGO***

1. Do you have animal housing for all animal species?/ *Mufite inzu zagenewe amatungo yose?*

| Day | Night |  |
| --- | --- | --- |
|  |  | Yes/ *Yego* |
|  |  | No/ *Oya* |

1. If yes, skip this question. If no, which animal species do not have housing?/ *Ni ba igisubizo ari yego, jya ku kindi kibazo. Ni ba igisubizo ari hoya, ni ayahe matungo adafite inzu yabugenewe?*

| **Time** | | **Species** | Where do the animals that do not have housing stay/live ?/ *Amatungo adafite amazu yabigenewe aba he ?* | | | |
| --- | --- | --- | --- | --- | --- | --- |
| **Day** | **Night** |  | **Kitchen**  ***Igikoni*** | **Storage room**  ***Aho mubika ibikoresho byo rugo*** | **People housing**  ***Inzu nini abantu babamo*** | **Other (specify)/ *Ahandi*** |
|  |  | Cattle |  |  |  |  |
|  |  | Goats/ sheep |  |  |  |  |
|  |  | Pigs |  |  |  |  |
|  |  | Poultry |  |  |  |  |
|  |  | Rabbits |  |  |  |  |
|  |  | Dogs |  |  |  |  |
|  |  | Cats |  |  |  |  |
|  |  | Others(species)  ____________ |  |  |  |  |
|  |  | Others(species)  ____________ |  |  |  |  |

1. Do you separate all animal species? / *Mutandukanya amatungo y’ubwoko butandukanye*?

|  | Yes/ *Yego* |
| --- | --- |
|  | No/ *Oya* |

1. If not, which animal species are kept together?/ *Niba mutayatandukanya, ni ayahe matungo abana n’ayandi?*
2. *:*_______________________________________________________________________________________
3. *:*_______________________________________________________________________________________
4. *:*_______________________________________________________________________________________
5. Do you separate animals of the same species by age categories/ *Mutandukanya amatungo y’ubwoko bumwe ari mu byiciro bitandukanye?*

|  | Yes/ *Yego* |
| --- | --- |
|  | No/ *Oya* |

1. Do you separate sick and healthy animals?/ *Mutandukanya amatungo arwaye n’atarwaye ?*

|  | Yes/ *Yego* |
| --- | --- |
|  | No/ *Oya* |

1. Do you isolate newly introduced animals? / *Amatungo yinjiye bwa mbere mu rwuri rwanyu mubanza kuyashyira ukwayo?*

|  | Yes/ *Yego* |
| --- | --- |
|  | No/ *Oya* |

1. If yes, how many days do you isolate them?/ *Niba mubikora muyamaza igihe kingana iki ari ukwayo?* …………………

| **Species** | **Time** |
| --- | --- |
| Cattle |  |
| Goats/ sheep |  |
| Pigs |  |
| Poultry |  |
| Rabbits |  |
| Dogs |  |
| Cats |  |
| Others(species) _________ |  |

1. Do you separate animal and human equipment/ utensils?/ *Mutandukanya ibikoresho by’abantu n’ibyo mukoresha mu bworozi?*

|  | Yes/ *Yego* |
| --- | --- |
|  | No/ *Oya* |

1. If no, which equipment/ utensils are shared with animals?/ Niba mutabitandukanya, ni ibihe bikoresho byo mu rugo amatungo ahuriraho n’abantu

|  | Drinkers |
| --- | --- |
|  | Feeders |
|  | Other (specify)/ *Ibindi___________________* |

1. Do children interact with animals?/ *Abana bajya bakina n’amatungo?*

|  | Yes/ *Yego* |
| --- | --- |
|  | No/ *Oya* |

1. If yes, which species?/ *Niba igisubizo ari yego, ni ayahe matungo?________________________________________________________________________________________________________________________________________________________________________________________________*
2. If you have cattle, do you use natural or artificial insemination? / *Niba mufite inka, murabanguriza cyangwa muteza intanga?*

|  | Natural/ *Kubanguriza* |
| --- | --- |
|  | Artificial insemination/ *Guteza intanga*. |

1. Do you assist animals in parturition? *Mujya mufasha amatungo yanyu iyo ari kubyara?*

|  | Yes/ *Yego* |
| --- | --- |
|  | No/ *Oya* |

1. If yes, do you use PPE while assisting animals in parturition? *Mwambara ibibakingira iyo muri kubyaza amatungo yanyu?*

|  | Yes/ *Yego* |
| --- | --- |
|  | No/ *Oya* |

1. Did you experience abortion in your farm? *Mwari mwagira itungo riramburura ?*

|  | Yes/ *Yego* |
| --- | --- |
|  | No/ *Oya* |

1. When you experience abortion, how do you discard the abortion tissues? *Ni he mushyira imyanda yavuye mu kuramburura*?

|  | Burn them/ *Murayitwika* |
| --- | --- |
|  | Bury them/*Murayitaba* |
|  | Put in normal trash/ *Mushyira mu kimoteri gisanzwe* |
|  | Other (specify)/ *Ibindi (sobanura)* |

1. What type of water do you drink?/ *Ni ayahe mazi munywa?*

|  | Mineral/ *Amazi yatunganyirijwe mu ruganda* |  |
| --- | --- | --- |
|  | Tape water/ *Amazi ya robine* |  |
|  | Lake water/ *Amazi y’ikiyaga* |  |
|  | Other (specify)/ *Andi* ____________ |  |

1. If you don’t drink mineral water, do you boil it before drinking?/ *Niba mutanywa amazi yatunganyijwe n’uruganda, mubanza kuyateka mbere yo kuyanywa?*

|  | Yes/ *Yego* |
| --- | --- |
|  | No/ *Oya* |

1. Do you boil milk before drinking?/ *Muteka amata mbere yo kuyanywa?*

|  | Yes/ *Yego* |
| --- | --- |
|  | No/ *Oya* |
|  | Not always/ *Si buri gihe* |

1. Do you eat undercooked meat?/ *Mujya murya inyama zidahiye neza ?*

|  | Yes/ *Yego* |
| --- | --- |
|  | No/ *Oya* |

1. Do you eat raw vegetables and fruits?/ *Mujya murya imboga n’imbuto bidatetse ?*

|  | Yes/ *Yego* |
| --- | --- |
|  | No/ *Oya* |

1. If yes, do you wash them before consumption? *Mwoza imboga n’imbuto mbere yo kuzirya?*

|  | Yes/ *Yego* |
| --- | --- |
|  | No/ *Oya* |

1. Do you use animal manure your crops?/ *Mujya mukoresha ifumbire yavuye ku matungo mu mirima yanyu?*

|  | Yes/ *Yego* |
| --- | --- |
|  | No/ *Oya* |

1. Do you use PPEs when handling manure?
2. What kind of crops?/ *Muyikoresha ku bihe bihingwa?*

|  | Vegetables/ *Imboga* |
| --- | --- |
|  | Fruits/ *Imbuto* |
|  | Maize/ *Ibigori* |
|  | Others (specify)/ *Ibindi* |

1. Do you use mosquito nets?/ *Murara mu nzitiramibu?*

|  | Yes/ *Yego* |
| --- | --- |
|  | No/ *Oya* |

1. Are there bushes around the farm?/ *Hari ibihuru hafi y’aho muba?*

|  | Yes/ *Yego* |
| --- | --- |
|  | No/ *Oya* |

1. How far is a natural water body?/ *Muturiye ikiyaga/ igishanga/ umugezi utemba ? ……………..*
2. Have your animals ever been vaccinated?

|  | Yes/ *Yego* |
| --- | --- |
|  | No/ *Oya* |

1. If yes, which diseases do you vaccinate for ? /*Ni izihe ndwara mukingira?*

| Species/*Ubwoko* | Disease/ *Indwara* | | When was the last time did you vaccinate?/ *Ni ryari uheruka gukingiza?* | Do you remember the date for the next shot? *Ni ryari uzongera gukingiza?* |
| --- | --- | --- | --- | --- |
| Cattle/ *Inka* | Anthrax & Black quarter/ *Ubutaka* |  |  |  |
|  | Brucellosis/ *Amakore* |  |  |  |
|  | Theileriosis/ *Teleriozisi* |  |  |  |
|  | Rift Valley fever/ *Ubuganga bwo mu kibaya cya Rift* |  |  |  |
| Goats & Sheep/ *Ihene n’intama* | Peste des Petit Ruminants (PPR)/ *Icyorezo cy’amatungo magufi* |  |  |  |
| Pigs/ *Ingurube* | Swine Erysipelas/ *Rouge* |  |  |  |
|  | African Swine Fever/ |  |  |  |
|  | Leptospiross/ |  |  |  |
| Poultry/ *Inkoko* | Infectious Bronchitis |  |  |  |
|  | Newcastle |  |  |  |
|  | Gumboro |  |  |  |
|  | Marek |  |  |  |
|  | Fowl pox |  |  |  |
| Dogs & Cats/ *Imbwa n’injangwe* | Rabies/ *Ibisazi* |  |  |  |

1. If no, why didn’t you vaccinate?/ *Niba mutabikora biterwa n’iki?*

|  | Didn’t know it was important |
| --- | --- |
|  | No information / *Nta makuru* |
|  | No access to vaccines/ *Inkingo ntiziboneka* |
|  | High prices of vaccines/ *Inkingo zirahenda* |
|  | Others (specify)/ *Ibindi* ________________ |

1. Do you have roaming animals in the neighbourhood that can have access to your farm?/ *Izi nyamaswa zijya ziza aho mutuye?*

|  | Dogs/ *Imbwa* |
| --- | --- |
|  | Cats/ *Ipusi* |
|  | Rats/ *Imbeba* |
|  | Monkeys/ *Inkende* |
|  | Bats/ *Uducurama* |
|  | Others (specify)/*Izindi* ____________________ |

**THANK YOU FOR PATIENCE/ *MURAKOZE KUTWIHANGANIRA*!!!**

Ending time/ *Isaha ibazwa ryarangiriyeho*: ______:________
